# Management and Thinking on the Treatment of Cancer Patients During the COVID-19

**DOI:** 10.3389/fmolb.2021.673360

**Published:** 2021-07-01

**Authors:** Shuangyue Pan, Jiahong Jiang, Zheling Chen, Liu Yang

**Affiliations:** ^1^The Second Clinical Medical College, Zhejiang Chinese Medical University, Hangzhou, China; ^2^Center of Oncology, Department of Medical Oncology, Zhejiang Provincial People’s Hospital, People’s Hospital of Hangzhou Medical College, Hangzhou, China

**Keywords:** COVID-19, SARS-CoV-2, management, cancer patients, vaccine, cancer immunity

## Abstract

Coronavirus disease-2019 (COVID-19) has spread rapidly around the world and has become a public health emergency of international concern. The weekly epidemiological report issued by the WHO pointed out that new coronavirus variants have appeared in 131 countries and regions, which demonstrates that the current epidemic situation is still severe. As of now, the severe acute respiratory syndrome coronavirus (SARS-CoV-2) has been widespread worldwide for more than one year and poses a serious threat to the health of vulnerable groups such as those with malignancies, the elderly, and the immunocompromised. Compared with the general population, cancer patients with COVID-19 infection are more likely to have serious clinical adverse events, leading to higher mortality. There is no doubt that during the COVID-19 epidemic, whether it is with regards to how to prevent infection or how to continue anti-tumor treatment, cancer patients are in a difficult situation. Meanwhile, an international patient with malignant Hodgkin’s lymphoma who was cured after being infected with the new coronavirus surprised us, and it inspires more scientists to explore the relationship between infection, immunity, and tumors. Relevantly, through multi-disciplinary discussion, scientists put forward more new perspectives on the treatment of future tumors and the management of SARS-CoV-2 diseases. In this review, the impact of COVID-19 on cancer patients is discussed in detail and the recommendations for the diagnosis, treatment and management of cancer patients will be put forward under the challenge of the COVID-19 epidemic. Furthermore, the safety and effectiveness of the SARS-CoV-2 vaccine will be discussed, and we will also put forward our insights on cancer immunity.

## Introduction

The novel coronavirus disease 2019 (COVID-19) caused by the severe acute respiratory syndrome coronavirus named SARS-CoV-2 occurred in Wuhan, Hubei in December 2019 and has now spread globally (Yu et al., 2020). SARS-CoV-2 virus is an enveloped positive single-stranded RNA virus that can infect both humans and animals (Velavan and Meyer, 2020). Human-to-human transmission has been proven by the infection of 15 medical staff in a Wuhan hospital (Paules et al., 2020). As of Feb 7, 2021, the total number of confirmed cases was 106,277,685 worldwide, and the total number of deaths was 2,316,660 (No author). In a clinical study from China, 81% of the cases were classified as mild pneumonia; 14% of the cases had serious diseases such as dyspnea and decreased blood oxygen saturation and 5% of the cases had respiratory failure, septic shock, or multiple organ failure. Among them, the overall case fatality rate was 2.3% (1,023 of 44,672 confirmed cases). However, the case fatality rate of patients aged 80 and over has increased to 14.8% (The Novel Coronavirus Pneumonia Emergency Response Epidemiology Team, 2020).

Cancer patients are commonly in a state of systemic immunosuppression due to anti-tumor therapies (such as chemotherapy or radiotherapy), so they are more susceptible to be infected with the SARS-CoV-2 virus (Liang et al., 2020). Nevertheless, anti-tumor therapy is essential for cancer patients to control their condition. Therefore, it is challenging to strengthen the management of anti-tumor treatment while preventing infection. Recently, the vaccination of the SARS-CoV-2 virus has given us full confidence in defeating COVID-19, and the case of a tumor patient cured after being infected with the SARS-CoV-2 virus has triggered our thinking.

This review aims to discuss approaches of management for cancer patients during the pandemic and provide our insights on the cure of tumor patient infected with the SARS-CoV-2 virus.

## Cancer Patients During the COVID-19

### Morbidity and Mortality of Cancer Patients

On the one hand, cancer patients are generally malnourished and have lower ability to fight SARS-CoV-2 virus; on the other hand, cancer patients have immunodeficiency due to the systematic treatment. These factors make cancer patients more susceptible to SARS-CoV-2 virus, and the mortality rate will be higher than normal (Wang and Zhang, 2020). A retrospective study in Zhongnan Hospital of Wuhan University showed that the infection rate of SARS-CoV-2 in cancer patients is 0.79% (12 of 1,524 patients; 95%CI; 0.3–1.2%). However, the cumulative incidence of all COVID-19 cases reported in Wuhan is 0.37% (41,152 of 11,081,000 cases; data cut off on February 17, 2020) (Yu et al., 2020). Cancer patients infected with the SARS-CoV-2 virus have more risk factors than patients without cancer (Heathcote et al., 2020). A study collected and analyzed 2007 confirmed cases of COVID-19 in 575 hospitals in China, of which 1,590 cases of infection are valid data. The study found that patients with cancer are older than patients without cancer (average age 63.1 years old [SD 12.1] vs 48.7 years old [16.2]), more likely to have a history of smoking (four [22%] of 18 patients vs 107 [7%] of 1,572 patients), had more polypnea (eight [47%] of 17 patients vs 323 [23%] of 1,377 patients), and more severe baseline CT manifestation (17 [94%] of 18 patients vs 1,113 [71%] of 1,572 patients) (Liang et al., 2020). These risk factors directly cause the mortality of cancer patients infected with the SARS-CoV-2 virus to be higher than that of the healthy population. A multi-centural study including 105 cancer patients and 536 age-matched non-cancer patients confirmed with COVID-19 showed that compared with COVID-19 patients without cancer, cancer patients have higher observed mortality (OR 2.34, 95% CI [1.15, 4.77]; *p* = 0.03), and a higher ICU hospitalization rate (OR 2.84, 95%) CI [1.59, 5.08]; *p* < 0.01) (Dai et al., 2020).

### Differentiation of Cancer Patients and COVID-19

Cancer patients have complex conditions, with many non-specific clinical symptoms and signs. For example, lung cancer patients’ own lung manifestations, tumor fever, immune-related pneumonia, and other manifestations. During this special period, it is necessary to actively differentiate from COVID-19. Multiple studies have shown that the main clinical manifestations of COVID-19 are fever, cough, and dyspnea (Huang et al., 2020; Jiang et al., 2020; Zhang L. et al., 2020). Although non-cancer and cancer patients with COVID-19 have similar clinical manifestations, a study screened 13,077 SARS-CoV-2 patients and finally compared the clinical characteristics of 232 cancer patients and 519 matched cancer-free patients. Studies have shown that cancer patients infected with the SARS-CoV-2 virus were more likely to have dyspnea and CT scans showed that ground-glass opacity and patchy opacity were more common. Furthermore, the pro-inflammatory cytokines of cancer patients including TNF-α, IL-6, and IL-2R were higher than those of patients without cancer. The lymphocytes, CD4+ T cells, CD8+ T cell counts, and the ratio of CD4+ T cells to CD8+ T cells in cancer patients decreased more significantly (Tian et al., 2020). Special attention should be paid to the appearance of any of these characteristics to minimize the risk of underdiagnosing cancer patients with COVID-19 infection.

In general, the fever of tumor patients can be divided into tumor fever, drug fever, and infection. Tumor fever refers to the response of the immune system to tumor necrosis and stress factors produced by tumors which is generally ≤38.5°C with no inducement and lasts for a long time. Experimental treatment with antibiotics is ineffective (Pasikhova et al., 2017). Drug fever means that fever occurs during medication and stops after drug withdrawal. The median time of fever caused by antineoplastic drugs is about 0.5 days after administration (Patel and Gallagher, 2010). Fever caused by infection usually has a higher temperature and can be reflected by blood routine tests.

**FIGURE 1:**
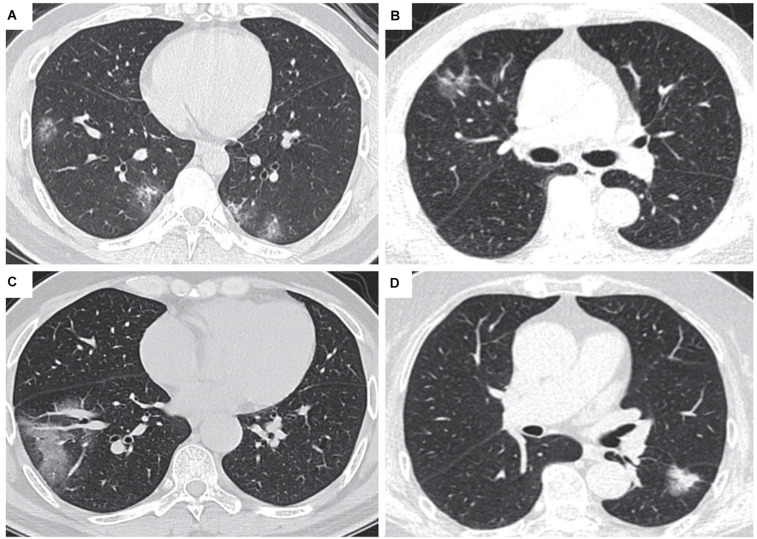
CT images of lung cancer patients and COVID-19 (Transl Lung Cancer Res.2020 Aug;9(4):1516-1527. doi: 10.21037/tlcr-20-892). **(A)** CT scan of a patient diagnosed with COVID-19: The lesion is GGO, and there is no sign of pleural retraction or vascular convergence. **(B)** CT scan of a patient diagnosed with lung cancer, with pleural retraction and cystic change. **(C)** CT scan of a patient diagnosed with COVID-19, the lesion showed patchy turbidity, irregular shape, and no pleural retraction. **(D)** CT scan of a patient diagnosed with lung cancer, with pleural retraction, lobulated sign, and spiculate protuberance.

All patients diagnosed with COVID-19 have varying degrees of lung abnormalities, which can be seen in chest computed tomography (CT) imaging (Huang et al., 2020; Figure 1). Most cases of COVID-19 showed bilateral parenchymal ground-glass opacity (GGO) or consolidative pulmonary opacity on CT scans, and the enlargement and consolidation of GGO usually indicate the progression of COVID-19 (Chung et al., 2020). The characteristics of COVID-19 also include reticular pattern, linear subpleural opacity, bronchial dilatation, centrilobular nodules and tree-in-bud (Zhang Y. J. et al., 2020). Through the imaging performance of CT, we can distinguish lung cancer from COVID-19 through the following points. First of all, most COVID-19 patients have bilateral lesions while most cancer patients have unilateral lung lesions. Secondly, most COVID-19 patients have more than one type of lung lesion, while lung cancer patients tend to have either pure GGO or mixed GGO. What is more, COVID-19 shows more patchy lesions, while lung cancer shows more oval lesions (Zhang Y. J. et al., 2020; Table 1). There are many types of lung cancer that various imaging manifestations may reveal, therefore, we need to use RT-PCR and next-generation sequencing methods applied to respiratory tract specimens to assist identification when necessary.

**TABLE 1:** CT differentiation of lung cancer and COVID-19.

	Lung cancer	COVID-19
Lesion(in most cases)	Unilateral	Bilateral
Lesion form	Oval lesions	Patchy lesions
Involved lobes	Less	More
Types of lung disease	Pure GGO or mixed GGO	More than one
Characteristic performance	lobulated signs, pleural retraction, cystic changes and signs of vascular convergence	Air bronchography, reticular pattern, subpleural linear opacity, bronchiectasis, lobular nodules and tree-in-bud

### Is Anti-tumor Treatment a Contraindication During the COVID-19?

Cancer patients are at increased risk of death and serious clinical events due to COVID-19 infection. However, risk of adverse events does not seem to be increased by cancer therapies (Heathcote et al., 2020). Between April 15 and 26, 2020, a total of 1,227 tumor patients were tested for SARS-CoV-2 using RT-qPCR in the tumor clinic, and 78 (6.4%) were positive. Among them, 75 (96.2%) were asymptomatic infections. Fourteen cancer patients out of 75 asymptomatic infections received chemotherapy or immunotherapy (±4 weeks of SARS-CoV-2 test), 48 (61.5%) of 78 patients who tested positive received glucocorticoid combination therapy. None of the patients with asymptomatic infection had unexpected complications caused by SARS-CoV-2 infection. These data indicate that the incidence of symptoms due to COVID-19 is relatively low among patients treated with chemotherapy and other immunosuppressive agents (such as glucocorticoids). Therefore, whether anti-tumor therapy is a contraindication during the epidemic is still inconclusive (Hempel et al., 2020).

**FIGURE 2:**
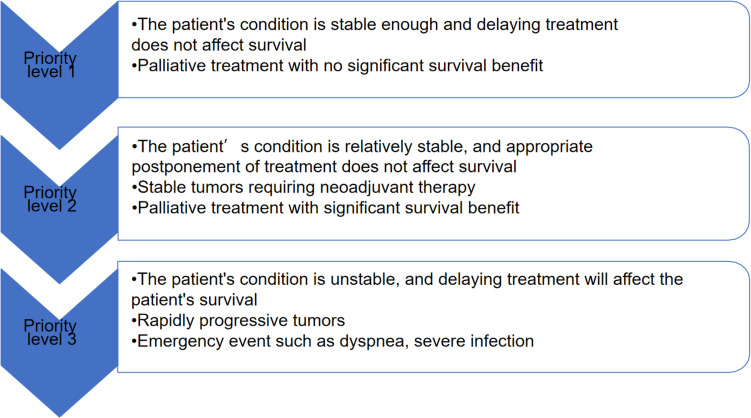
Classified treatment management according to the actual situation of cancer patients.

Studies have shown that the risk of death in patients infected with COVID-19 is significantly related to the increase in patient age (odds ratio 9.42 [95% CI 6.56–10.02]; *p* < 0.0001) and significant correlation with other comorbidities, such as hypertension (1.95 [1.36–2.80]; *p* < 0.001) and cardiovascular disease (2.32 [1.47–3.64]). Anti-tumor treatment does not increase the risk of death from new coronary pneumonia in cancer patients (Lee et al., 2020). Therefore, for cancer patients, risk-benefit assessment must be carried out. If the benefits outweigh the risks, cancer treatment should continue (Gosain et al., 2020; Zhao Q. et al., 2020; Figure 2).

Patients at the priority level 1 do not need to go to the hospital for treatment unless necessary, and can communicate with doctors through telemedicine and other methods; Patients at the priority level 2 can delay treatment appropriately; patients at the priority level 3 should go to the hospital for treatment with protective measures.

### Cancer Patients With Different Treatment Modalities

#### Cancer Patients Undergoing Surgery

During the COVID-19 pandemic, patients with tumors requiring surgery should be strictly screened for infection with the SARS-CoV-2 Virus, and a comprehensive assessment should be made based on the patient’s overall condition as to whether surgery can be performed to avoid serious adverse events. An international study conducted in 235 hospitals in 24 countries/regions included all surgical patients who were confirmed to have SARS-CoV-2 infection. The primary outcome measure was the 30-day mortality rate after surgery, and the secondary outcome measure was pneumonia, acute respiratory distress syndrome, or accidental ventilation after surgery. Among 1,128 patients undergoing surgery, 294 patients (26.1%) were confirmed to have SARS-CoV-2 infection before surgery, their 30-day mortality rate was 23.8% (268 out of 1,128). Pulmonary complications occurred in 577 (51.2%) of 1,128 patients. The 30-day mortality rate of these patients was 38% (219 of 577), accounting for 82.6% of all deaths (219 of 265) (Nepogodiev et al., 2020). Therefore, it is necessary to carefully consider the surgical operation of cancer patients during the epidemic.

If cancer patients do not receive surgery in time, how long will the extension affect the survival rate or the probability of complete resection? Studies have shown that for cancers treated with surgery first, the median safe postponement period (SPP) is 3 weeks which is 6 weeks from diagnosis. For 48% of cancer types, the SPP was at least 4 weeks. For patients who received neoadjuvant therapy such as chemotherapy or radiotherapy, the median SPP was 8 weeks which is 26 weeks from diagnosis. For 76% of cancer types, the SPP is at least 6 weeks (Kiran and Turaga, 2020). Through this study, we can find that most cancer operations can be postponed for at least 4 weeks without significantly affecting the survival of the patient. The tumor will not progress significantly, and there is no significant difference in the probability of complete tumor resection.

#### Cancer Patients Undergoing Routine Chemotherapy

During the COVID-19 pandemic, the main concern of patients receiving chemotherapy was the decrease in anti-tumor efficacy due to the interruption of chemotherapy. While we pay attention to the adverse effects of the epidemic on the delay of chemotherapy for tumor patients, we should also treat the adjustment of chemotherapy regimens rationally (Zhao Z. et al., 2020). Based on the fact that anti-cancer therapy may bring greater risks, some patients may voluntarily choose treatments with lower potential efficacy but a lower degree of myelosuppression (Battershill, 2006). Patients who receive conventional chemotherapy can consider switching from intravenous chemotherapy to oral anticancer drugs, which can reduce the number of visits to the hospital and thus reduce the risk of infection (Gosain et al., 2020). In addition, with the advent of elastic pumps, chemotherapy at home has become more and more common (Shereen and Salman, 2019). In order to reduce the duration of exposure in the hospital, the hospital pharmacy department can deliver medicine to patients by express delivery (Willan et al., 2020). The treatment of lung cancer patients during the COVID-19 pandemic can give us some suggestions: some scholars have proposed that multiple variables should be considered when formulating a diagnosis and treatment plan for lung cancer patients, including virus prevalence, capacity of local medical institutions, patient infection risk, cancer status, patient comorbidities, age, etc. After considering these factors, the treatment of lung cancer patients is divided into five groups “Should be started when Possible,” “Should not be stopped without justification,” “Can be given preferentially,” “Can be withheld or delayed after careful consideration,” “Should not be started without justification” (Banna et al., 2020). Under the guidance of oncologists, the treatment strategy should be adjusted appropriately according to the progress of tumor patients, the stage and effect of anti-tumor therapy, local epidemic situation, and so on (Zhao Z. et al., 2020).

#### Cancer Patients With Radiotherapy

Studies have shown that delayed radiotherapy and interruption of radiotherapy may contribute to inferior local control and overall survival of cancer patients (Yao et al., 2018). Radiotherapy requires specific equipment, and community hospitals have no extra resources to reserve linear accelerators, simulators or mold rooms for patients, the radiotherapy center cannot disperse patients, therefore it will be in a high-risk area (Mukherjee et al., 2003). During the outbreak of COVID-19 in Wuhan, the department of radiotherapy center has taken the following measures: divide the radiotherapy center into different infection control areas and provide different levels of protection; implement daily symptom testing standards for patients receiving treatment; design and implement a revised radiotherapy workflow, special treatment area cleaning and disinfection policies and procedures (Wei et al., 2020).

#### Cancer Patients Receiving Immunotherapy

Immune checkpoint inhibitors play an anti-tumor effect by regulating the body’s own immune response. Checkpoint antibody inhibitors, such as anti-PD-1/PD-L1, are novel inhibitors that function as tumor suppressors by regulating immune cell-tumor cell interaction (Alsaab et al., 2017). The research team analyzed a total of 800 patients with COVID-19 diagnosed with cancer and symptoms. Among them, 412 (52%) patients had mild COVID-19 symptoms; eventually 226 patients (28%) died. The research team found that in the past 4 weeks, patients using immunotherapy, hormone therapy, targeted therapy, and radiation therapy had no significant impact on mortality (Lee et al., 2020). However, research results published by the famous Memorial Sloan-Kettering Cancer Center (MSKCC) in New York, United States revealed that cancer patients who use immunotherapy (mainly PD-1/PD-L1 inhibitors) were more dangerous after infection with SARS-CoV-2 virus and required a higher proportion of hospitalization, the rate of severe pneumonia was also higher (Robilotti et al., 2020). Cancer immunotherapy can effectively prolong the survival period of cancer patients but also cause some adverse reactions such as organ inflammation, the most common of which is immune-related pneumonia. Distinguishing between COVID-19 pneumonia and immune-related pneumonia is a diagnostic challenge (Suresh et al., 2018). The clinical manifestations of immune-related pneumonia and COVID-19 are similar and they can both show symmetrical patchy GGO and consolidation area in CT imaging. Therefore, a diagnostic algorithm is proposed to distinguish immune-related pneumonia and COVID-19. The first step is RT-PCR screening. Secondly, laboratory examination and CT imaging features are helpful for identification. There is no obvious specificity in the hemogram of immune-related pneumonia, just CRP and ESR are often increased. In the early stage of COVID-19 patients, peripheral blood leukocytes were normal or decreased, lymphocyte count decreased, CRP and ESR increased in most patients, D-dimer and liver enzymes, LDH, muscle enzymes, myoglobin, and troponin increased in severe patients (Kattan et al., 2020; Wu et al., 2020). Besides, further bronchoscopy is needed if necessary (Dumoulin et al., 2020).

#### Cancer Patients Participating in Clinical Trials

During the COVID-19 period, individualized management of clinical trials should be carried out to maximize the protection of patients’ interests. Effectively guard against COVID-19 and ensure the scientific nature of clinical experiments at the same time. The Food and Drug Administration (FDA) has issued guidelines for institutions to protect trial participants and administer investigational products by changing monitoring methods (FDA, 2020). The progress of clinical trials may be slowed down due to the impact of the epidemic, because clinical trial subjects have strict standards and time limits for drug administration, efficacy evaluation, safety evaluation, follow-up, and so on. It requires the cooperation of subjects, researchers, sponsors, clinical research coordinators, and other relevant participants under the supervision of ethics. The clinical trial activities during the COVID-19 period should be tailored to the changing epidemic situation. Suspension of some non-therapeutic intervention trials could be considered, and clinical trials that continue after discussion will continue to obtain all the tests and data points needed for the study (Gosain et al., 2020). The researchers can manage according to the different dosage forms used by the subjects, subjects taking oral drugs can be followed up remotely by telephone or Internet, and the researchers can send the drugs to the subjects by express delivery.

## Cancer Patients Infected With SARS-CoV-2 Virus

### A Patient Diagnosed With Malignant Hodgkin’s Lymphoma Was Cured After Being Infected With the SARS-CoV-2 Virus

Recently, two British doctors reported a special case: a 61-year-old man diagnosed with Hodgkin’s lymphoma [EBV viral polymerase chain reaction (PCR) 4,800 copies/ml; log^10^3.68] infected with SARS-CoV-2 virus and was not given corticosteroids and immunochemotherapy. After 4 months, PET CT showed widespread resolution of the lymphadenopathy and the EBV viral PCR had also fallen to 413 copies/ml (log^10^2.62) (Challenor and Tucker, 2021). Researchers suspected that the SARS-CoV-2 virus stimulated an anti-tumor immune response, killing the virus while also killing cancer cells. This is not the only case that the tumor is cured after infection. The case in 2012 reported that a 67-year-old woman with lymphoma infected with pneumonia and colitis and the tumor also disappeared completely (Buckner et al., 2012). Although lymphoma does not represent all tumors, it does give us a great inspiration. In fact, there have been similar records dating back hundreds of years. Some doctors even tried to inject pathogens into cancer patients to induce fever to treat cancer. This is actually the original form of immunotherapy. In recent years, PD-1 inhibitors, CTLA-4 inhibitors, CAR-T therapy, CAR-NK therapy, and other modern immunotherapies have been successful, bringing hope to many cancer patients. The idea that activating the immune system can fight cancer has been widely accepted.

### The Contradiction Between the Treatment of Infectious Diseases and Anti-tumor Treatment

Infection is one of the most common complications of anti-tumor therapy. Anti-tumor treatment destroys the patients’ immune system, resulting in neutropenia which can lead to more severe infection. The use of antiviral therapy in cancer patients infected with COVID-19 remains controversial. Among cancer patients infected with COVID-19 in a case report, 20 patients (71.4%) were treated with antiviral drugs empirically; 9 patients (32.1%) received a combination of antiviral drugs, 15 patients (53.6%) received systemic corticosteroid therapy, 12 patients (35.7%) received intravenous immunoglobulin. The data from the case did not report that the patient population had benefited from it. Instead, of the 6 cancer patients who received anti-tumor treatment within 14 days of being diagnosed with COVID-19, 5 (83%) had serious incidents (Zhang L. et al., 2020). Although the available information is very limited, it is not recommended to use anticancer drugs and antiviral treatments at the same time outside of clinical trials to avoid unexpected pharmacokinetic interactions and toxicity (Raymond et al., 2020).

### The Impact of Infection on Tumor Prognosis

As mentioned above, a patient with lymphoma had tumor regression after being infected with the SARS-CoV-2 virus. The case of cancer patients whose tumor disappeared after infection has been reported many times. A review of past reports found that self-healing was usually related to acute infections such as bacteria and viruses, fever, and immune stimulation (Buckner et al., 2012; Challenor and Tucker, 2021). The idea of stimulating the human immune response to treat cancer and other diseases has been proven in trials.

Vaccines are a typical example of preventing the invasion of microorganisms by stimulating the human immune response. Bacille Calmette-Guerin (BCG), a live attenuated strain of Mycobacterium bovis, is the first live-attenuated vaccines used in humans. BCG provides 80% protection against severe and disseminated tuberculosis in children and can also reduce the risk of adult tuberculosis (Ritz et al., 2008). In the 1980s, BCG became the first choice for the treatment of early *in situ* bladder cancer. As long as the patient’s immune system is normal, the tumor burden is small, the BCG vaccine can directly contact the tumor, and the dosage of the drug is sufficient, the BCG vaccine can eliminate the tumor in 70% of patients (Babaian, 2021). Researchers have found that urothelial cells and immune system cells both play a crucial role in the anti-tumor treatment of BCG. BCG vaccine can stimulate the body’s immune response through its internalization, bladder cancer cells up-regulate the expression of MHC class II and ICAM-1 and secrete various cytokines, including IL-6. In addition to bladder cancer cells, dendritic cells may also play a role in the recruitment of immune cells, initially granulocytes, then macrophages and lymphocytes (Redelman-Sidi et al., 2014).

In addition to BCG, oncolytic viruses can achieve anti-tumor effects through the dual mechanism of selective killing of tumor cells and inducing systemic anti-tumor immunity. Oncolytic viruses are attenuated viruses that infect tumor cells to enhance the body’s natural immune response. Oncolytic viruses have direct and indirect toxic effects on tumor cells, such as autolysis, honing of immune cells, destruction of vascular supply, and enhancement of other auxiliary anti-cancer therapies (Russell et al., 2012). Currently, the oncolytic virus treatment drug talimogene laherparepvec (T-Vec or Imlygic) for metastatic melanoma has been approved by the FDA.

Therefore, the pathogenic bacterium is not completely harmful to cancer patients, and there are many clinical applications that use the properties of pathogenic bacterium to bring cancer patients benefits such as the use of inactivated pathogenic bacterium to prepare vaccines; the use of genetically engineered viruses as carriers for gene therapy; using viruses to target cancer, etc.

## Cancer Patients and SARS-CoV-2 Vaccines

In order to control the spread of the SARS-CoV-2 virus in a timely and effective manner, researchers have made efforts to develop vaccines (Hodgson et al., 2021). On November 9, 2020, Pfizer and BioNTech announced that the vaccine BNT162b2 against COVID-19 had succeeded in the Phase 3 study. Interim analysis showed that compared with placebo, two vaccination of the mRNA vaccine at 21 days intervals can reduce the infection rate of the symptomatic new coronavirus SARS-CoV-2 by 90% (Pfizer, 2020). The other clinical trial in the United States showed that the effective rate of mRNA-1273 vaccine in preventing COVID-19 diseases was 94.1% (Baden et al., 2021). At present, five COVID-19 vaccines have been approved for marketing or emergency use in China, including three inactivated vaccines and one adenovirus vector vaccine. Vaccines are essential to prevent COVID-19 and to protect patients with high-risk complications. As of April 1, 2021, more than 590 million people have been vaccinated worldwide (Caixin, 2021).

Cancer patients and their families are very concerned about a question: Can cancer patients get the COVID-19 vaccine? Due to the low immune function of cancer patients, the live microorganisms in the live attenuated vaccines will increase the risk; the live vaccines may cause excessive immunity in patients receiving immunotherapy due to the activation of immunity in the body. Therefore, according to the latest NCCN guidelines, live attenuated vaccines cannot be used for tumor patients with weakened immune function (NCPGI, 2020). However, despite the fact that the vaccines marketed in China are inactivated, patients with malignant tumors are still listed as one of the contraindications due to lack of clinical data. Therefore, it is still necessary to strengthen the management of cancer patients for hospital departments and cancer patients should also pay attention to personal management.

## COVID-19 Prevention and Control Management in Hospital Departments

**FIGURE 3:**
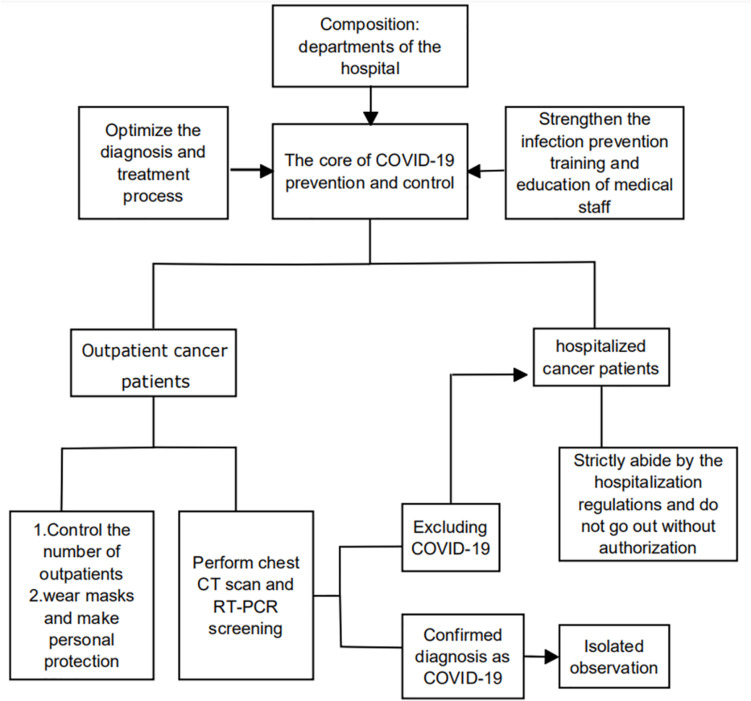
COVID-19 prevention and control management in hospital departments.

All departments of the hospital must provide patients with preventive and control measures, and each department must establish a COVID-19 prevention program within the department, which is composed of the core team of the department and the supervisors of each work link. It is essential to provide infection prevention training and education to all medical staff in the department and develop a clear process that everyone can strictly follow and implement (The Healthcare Infection Control Practices Advisory Committee [HICPAC], 2018). For outpatients, first of all, the number of outpatients should be controlled to the least level for reducing exposure to infection. Secondly, necessary temperature tests, emphasizing the importance of wearing masks and hand hygiene are effective measures to reduce infections (Al-Shamsi et al., 2020). It is recommended that qualified hospitals actively develop telehealth strategies to provide health care and reduce personnel gathering and unnecessary cross-infection (Centers for Disease Control and Prevention, 2020; Zhao Q. et al., 2020). For hospitalized cancer patients, strict screening must be carried out before anti-cancer treatment (Figure 3). Data from China shows that chest CT is more sensitive than RT-PCR for diagnosing COVID-19,therefore, the screening of the cancer patients for infection requires two examinations of chest CT scan and RT-PCR to consolidate the diagnosis (Ai et al., 2020).

## Personal Management and Social Assistance for Cancer Patients

Cancer patients should try to avoid going in and out of public places which is more likely to be exposed to SARS-CoV-2 virus, and ideally wear a mask when in close contact with others to prevent spread of respiratory secretions when they are coughing, sneezing, or talking (Centers for Disease Control and Prevention, 2020). Enhancing immunity and reducing the risk of exposure are important principles for cancer patients to prevent SARS-CoV-2 infections. Supplementation with some of dietary components such as dietary protein, omega-3 fatty acids, vitamin A can improve the conditions of cancer patients and enhance immunity (BourBour et al., 2020). Besides, the psychological bearing capacity of cancer patients is lower than that of normal people, coupled with social distance measures, isolation measures, and visitor restrictions, which limit the mental strength of cancer patients from family support (Al-Shamsi et al., 2020). We believe that while doing a good job in the prevention and control of the epidemic situation, we also need to pay attention to the mental health of tumor patients, and the research and application of related assessment, intervention and treatment measures is extremely urgent. Therefore, hospitals and medical workers should provide mental support to patients, and psychosocial workers should make maximum use of available resources to intervene patients to meet the continuous needs of patients and their families (Wang et al., 2020).

## Use Telemedicine to Achieve Full Management of Cancer Patients

Telemedicine has been shown to reduce medical costs and provide health care to populations with limited access to medical care (Sirintrapun and Lopez, 2020; Tashkandi et al., 2020). Examples of successful telemedicine in oncology include chemotherapy monitoring, symptom management, survival care, palliative care, and clinical trials (Sirintrapun and Lopez, 2020). It has been confirmed in several clinical trials that the outcome of telemedicine is similar to that of face-to-face care (Sirintrapun and Lopez, 2020). We can reduce the number of hospital visits during the COVID-19 pandemic by replacing some clinics with virtual clinics (via videoconferencing or telephone calls) (Tashkandi et al., 2020). In addition, we can also use store-and-forward methods, such as the short message service (SMS), email consultation, or regularly collect and upload data through networked devices to monitor symptoms and signs (McLean et al., 2013). Patients who do not have access to active treatment during the COVID-19 pandemic are particularly suitable for telemedicine. However, virtual telemedicine management is a developing tool to provide medical services to cancer patients under specific conditions. The main limitations of telemedicine include the jurisdiction of medical practice, restrictions on physical examination, and problems related to telemedicine reimbursement, etc. (Sirintrapun and Lopez, 2020).

## Conclusion

The COVID-19 pandemic has caused a huge public health crisis, which has brought tremendous pressure to medical staff and also brought unprecedented challenges to the treatment and management of cancer patients. This review provides detailed suggestions for dealing with COVID-19 from different aspects to help cancer patients affected by the epidemic. The development of the epidemic is very rapid, far beyond our imagination. Therefore, clinicians and medical personnel must strictly follow the epidemic prevention and control measures and modify or adjust the recommendations provided here as needed.

## Author Contributions

ZC and LY contributed to the conception and design of the work and data processing and analysis. ZC and SP performed the studies. ZC and SP prepared the majority of the manuscript and LY revised it critically. All authors have given final approvals to the version to be published.

## Conflict of Interest

The authors declare that the research was conducted in the absence of any commercial or financial relationships that could be construed as a potential conflict of interest.
